# The Economic and Psychological Impacts of COVID-19 Pandemic on Indian Migrant Workers in the Kingdom of Saudi Arabia

**DOI:** 10.3390/healthcare9091152

**Published:** 2021-09-03

**Authors:** Mohammed Arshad Khan, Md Imran Khan, Asheref Illiyan, Maysoon Khojah

**Affiliations:** 1 Accounting Department, College of Administrative and Financial Sciences, Saudi Electronic University, Riyadh 11673, Saudi Arabia; m.khoja@seu.edu.sa; 2 Department of Economics, Faculty of Social Sciences, Jamia Millia Islamia (A Central University), New Delhi 110025, India; imran738@gmail.com (M.I.K.); ailliyan@jmi.ac.in (A.I.)

**Keywords:** COVID-19, pandemic, migrants, sample survey, employment status, remittances, migrants’ perception, economic and psychological impacts

## Abstract

The ongoing Coronavirus disease 2019 (COVID-19) pandemic has changed the working environment, occupation, and living style of billions of people around the world. The severest impact of the coronavirus is on migrant communities; hence, it is relevant to assess the economic impact and mental status of the Indian migrants. This study is quantitative in nature and based on a sample survey of 180 migrant workers. Descriptive statistics, chi-square test, dependent sample *t*-test, and Pearson’s correlation coefficient were utilized to analyze the surveyed data. The findings of the study reveal, through the working experience of the migrants, that new international migration has reduced due to lockdown and international travel restrictions. It was also reported that the majority of the migrants worked less than the normal working hours during the lockdown, causing a reduction of salary and remittances. Chi-square test confirms that the perceptions of migrants towards the COVID-19 management by the government were significantly different in opinion by different occupation/profession. Majority of the sampled migrants reported the problem of nervousness, anxiety, and depression; however, they were also hopeful about the future. The psychological problem was severe for the migrants above the age of 40, not educated, and with a higher number of family members. Subsequently, the policy implications from the findings of the research can draw attention of the policy makers towards protective measures which need to be implemented to support migrants during the ongoing pandemic. The government should take some necessary steps, such as a financial benefit scheme, to overcome the problems in the reduction of migrant earnings and remittances. The government should not focus only on vaccination and physical fitness of the migrants but also need to find out the cure of the psychological impact arising during the pandemic.

## 1. Introduction

The dangerous ongoing pandemic, Coronavirus disease 2019 (COVID-19), reported in Wuhan city, China, in December 2019 and it is caused by a novel coronavirus called SARS coronavirus 2 (SARS-CoV-2) [1]. The director general of the World Health Organization (WHO) initially declared the spread of coronavirus as a public health emergency of international concern on 30 January 2020. Later on, the WHO declared a pandemic on 11 March 2020 [2]. The novel coronavirus is unique in nature because of high man-to-man transmission and has spread to 176 million people worldwide and caused 3.8 million deaths as of 15 June 2021 [3]. This pandemic has changed the occupation and living style of billions of people around the world and raised questions of medical facility arrangements of the different countries of the world. The government of China started imposing restrictions and the lockdown in Wuhan city began on 23 January 2020, followed by India on 24 March and Saudi Arabia on 25 March 2020 [4]. The main objective of the lockdown or curfew is to limit the spread of the virus by maintaining social distancing and creating medical facility on war footing. Lockdown; banned public gatherings; suspending religious activities; closure of business, schools, colleges, etc.; curfews; and restriction or suspension of all the travel domestically as well as internationally were followed by the majority of countries as preventative measures. The government of Saudi Arabia had suspended all its international flights on 15 March 2020 and resumes after 14 months on 17 May 2021; however, the suspension of flights will continue to 13 countries, including India due to the second wave of corona virus [5].

The COVID-19 pandemic is not only a health emergency but also a labor market and economic crisis because of its effects on the business status of millions of individuals. The Saudi health ministry took the initiative to provide free corona vaccine and also offered free corona screening and health care services to all of its citizen, including migrants workers, and made vaccine compulsory for the health care workers participating in Hajj and Umrah (Islamic pilgrimage to Mecca) initially and later on made it compulsory for all male and female private and public sector workers to attend the workplace [5,6,7]. COVID-19 immunization will be required to participate in any socio-cultural, commercial, economic, entertainment, or supporting affairs in Saudi Arabia from 1 August 2021 [7,8]. The government of Saudi Arabia is strict towards the enforcement of COVID-19 regulations to reduce its spread and violators are fined between Saudi Riyal (SAR) 10,000 to SAR 100,000; however, a second wave of COVID-19 hit the country in the beginning of February 2021 [9]. 

According to Indian Census-2011, India had 45.6 crore migrant population (38%) and, according to the recent report published by “United Nations Department of Economic and Social Affairs—2019”, India continues to have the maximum of its people (17.5 million) living overseas and highest remittance receiving country (USD 78.6 billion). Saudi Arabia is the third top remittances sending country (USD 36.1 billion) in the world and ranked third (13 million) in largest number of international migrants in the world. India–Saudi Arabia shifted from the tenth (2000–2010) to seventh largest bilateral migration corridor in the world [10,11].

The COVID-19 pandemic had reduced the new international migration and increased the returnee migrants, which happens to be the first time in recent history. According to an estimate by World Bank, a total of 6,000,000 migrants were evacuated through special flights (Vande Bharat Mission) and Kerala was affected the most by 4,000,000 returnee migrants. The estimated remittances (World Bank) to India will fall by 9% in 2020 and 14% in 2021 and the flow of foreign direct investment will fall by 36% in 2020; however, India will continue to be the top remittance recipient country globally, with approximately USD 76 billion which will be 2.9% of its Gross Domestic Product (GDP). The monetary emergency accentuated by COVID-19 could be long, profound, and inescapable when seen through a relocation focal point [12]. The oil rich country and job-rich sector in Saudi Arabia was drastically affected by Corona virus because of the drop in trade, disruption of production, tourism (Hajj and umrah), and hospitality. Lockdown and travel restriction reduce the demand for oil globally, and consequently oil prices had fallen by 50% in March 2020. To recuperate the economic slowdown, the Saudi government allowed private sector companies to cut the salaries of the workers up to 40% for a period of six month and thereafter could also terminate the contract [13,14]. The majority of the migrant workers in Saudi Arabia are engaged in the construction sector, agriculture, hospitality, and domestic work, which are highly affected by the ongoing pandemic. The acutely affected migrants in the state during the pandemic are domestic workers, low skilled/low-income workers, contract terminated or completed workers, informal workers, women migrant workers, and salaried employees. In this context, the present study attempts to make a deeper analysis of economic and psychological impacts of COVID-19 pandemic on Indian migrant workers in Saudi Arabia. The paper is coordinated as follows: Section 1 is introductory, Section 2 reviews the literature, Section 3 describes the research gap, Section 4 delineates the research methodology, and Section 5 details the results and findings of the study. The last section, i.e., Section 6, concludes the paper. The limitations and future scope for work are also described in this section.

## 2. Review of Literature

The Kingdom of Saudi Arabia identified its first Corona virus positive case in Qatif (Eastern Region): a person returned from Iran through Bahrain on 2 March 2020. The Saudi government reacted accordingly by limiting domestic travel, suspending the e-visa program, closing schools and colleges, closing non-essential industries, and imposing lockdown in Qatif region on 8 March 2020, followed by a temporary ban on international flights on 15 March and domestic flights on 21 March [15]. The Saudi government issued general guidelines as preventive measures to limit the spread of virus in the early stage by ensuring social distancing, measuring temperature before entering in public places, and mandatory of wearing face mask in public places. Lack of compliance was penalized with SAR 1000 (Saudi Riyal) [16].

The undocumented immigrants (with no legal rights to reside in the country) in any country are at higher risk during the pandemic, as they are probably not getting the relief benefits provided by the local government and are living in fear of deportation. The Saudi government has decided to provide proper health care services to undocumented migrants without any legal action or deportation; however, not everybody is enjoying the legal access to health care facilities due to fear of deportation [17,18].

The COVID-19 pandemic has influenced the worldwide economy as well as Saudi Kingdom’s economy. The most impacted sector, due to a ban on religious Hajj and Umrah, was the hospitality sector, especially in Makkah and Madinah. The International Air Transport Association estimates USD 7.2 billion loss of the Saudi aviation sector in 2020 due to suspension of international flights, which also affected the job status of 287 thousand people in this sector. Lockdown and suspension of international flights globally reduces the demand for oil to approximately 80 million barrel per day: consequently, the oil price was decreased by 58% in the beginning of 2020. The total decline in export of chemical and related industries due to blocked international trade were estimated to be more than SAR 10 billion in 2020 [19,20].

One study reveals that the wage cut among the gulf workers ranges between 25% to 50%, especially in education, hospitality, and other service-related industries, and income of foreign workers had substantially reduced (83% in Dubai and 35% in Jeddah) due to closure of industries as well as a drop in remittances to their respective countries by 44% during the initial wave of pandemic. Many migrants (from the Philippines, Pakistan, and Egypt) in Saudi Arabia do not want to return back to their respective countries because of the good health and emergency services in the destination country and poor medical services, high unemployment rate, and lower wage rate in their country of origin. In the same survey (N = 117), a majority of the studied migrants (89.7%) responded that they believe that gulf cities have effectively controlled the pandemic and 53% of respondents believe that locals’ attitudes towards the migrant did not change, 29.9% positively changed, and 17% reported negative changes in their attitude during the pandemic [20].

The world economy is suffering from economic slowdown and an unemployment crisis due to COVID-19. One research survey by APCO worldwide (The Association of Public-Safety Communications Officials) shows that 40% of Saudi citizens spend a lesser amount on the purchases of goods and services than before COVID-19; however, the majority of the respondents (81%) believe that the Kingdom will recover from the pandemic faster than other countries in the region because of the immediate action taken by the government [21]. In order to recover the economy from the pandemic and overcome the effect on private sector, the Saudi government implemented a relief package of USD 32 billion, which will help the hospitality sector to recover. The Kingdom of Saudi Arabia also focuses on the SME (small and medium enterprises) sector by announcing a financial support of SAR 50 billion. To manage the budgetary deficit raised because of the fall in petrochemical revenue, the Saudi government chose to expand the Value Added Tax (VAT) from 5 percent to 15 percent and also attempt to help the economy by implementing technology-based solution especially for the education sector and e-businesses [22].

Another study estimates a total 21% loss of the expected earning of low skilled Indian migrant workers in Saudi Arabia and a 36% loss adding recruitment cost and total remittances could fall by USD 2 billion due to COVID-19. This estimation was done by using simple estimation model and used the data collected by KNOMAD-ILO (The Global Knowledge Partnership on Migration and Development along with International Labor Organization) in 2016-17 [23].

Psychological impact (anxiety and depression) on well-being is normal during any pandemic outbreak. Coronavirus disease had to have severe psychological effects due to the uncertainty associated with it. It is important to focus attention on physical health along with measures to balance the mental status of the people. A sample study on psychological impact on the general population in the context of Saudi Arabia was carried out at the time of COVID-19 curfew and lockdown, and revealed that stress, anxiety, and depression were majorly found in medical workers, students, females, and persons with a mental disorder, and 1/4th of the sample population experienced moderate to serious psychological effects [24,25]. Another sample study for Saudi Arabia during the first wave of Corona virus disease stated that a marginal but substantial portion of the general population had found symptoms of anxiety and depression, and a majority of them reported symptoms of psychological distress [26]. The emotional wellbeing in any society is defined by measuring distress, depression, anxiety, and behavioral control among the people. The emotional wellbeing in the general population of Saudi Arabia was found to be moderate during the COVID-19 pandemic. It was positively affected because the health authority of the Saudi government responded in a timely manner and adopted effective measures to control the spread of the virus [27]. Job status of the workers (employed or unemployed) during the pandemic has drastically changed, which also influences the mental status of the people. The symptom of depression, as evidence from South Africa suggests, was less frequent among individuals who retained paid employment during lockdown than those who lost their job [28].

The migrants in the society are more prone to have psychological distress during any outbreak because of the job insecurity and loneliness. A study assessing the psychological impact on Indian migrant-workers during COVID-19 lockdown reveals that the symptoms of anxiety and depression are severe in migrant workers. A majority of the respondents (73.5%) reported the symptom of depression, half of them were positive for anxiety, and nearly 51% of the migrant workers were found to have the symptom of both anxiety and depression [29]. Another study about the low wage migrant workers in Singapore during the pandemic revealed they are at higher risk of bearing significant health, mental, and socio-economic effects [30]. A study was carried out to understand the prevalence of anxiety, stress, and depression among repatriated Indonesian migrant workers during coronavirus pandemic. It was found that symptoms of anxiety, stress and depression were somewhat lower when contrasted with overall public and medical care in the country. The risk of anxiety and depression were found to be low in educated, young, and married people. The risk is higher among the people who had negative perceptions about the wellbeing and COVID sicknesses. Better health care services and improved quarantine facilities were found to be crucial to reduce psychological problems of repatriated migrant workers [31].

## 3. Research Gap

To date, many studies have considered the socio-economic impacts of Corona virus on the general population, ignoring the economic impacts on migrants and their perceptions of the COVID-19 management by the government. Several studies also assess the psychological or mental effect of Corona virus on the general populace of the country; however, in the current paper we also analyze the psychological impact on the migrants.

## 4. Research Methodology

The present study assesses the economic and psychological impacts on Indian migrant workers in the Kingdom of Saudi Arabia during the Corona virus pandemic. This study is quantitative in nature and depends on a sample-survey approach. Both primary and secondary information are utilized. Primary data were collected through a process of structured Google Forms questionnaire. The respondents of this study were Indian migrant workers in Saudi Arabia who were selected through non-probability snowball sampling techniques. This technique of data collection was used so that researchers could identify the Indian migrants in the Saudi Arabia due to COVID-19 related restrictions. The survey was in English language and titled as ‘COVID-19 Pandemic and Indian Migrants in Saudi Arabia’, and data were collected by sharing the link of the Google form through different social networking sites for literate migrants and through telephonic interview for illiterate migrants. The survey contained 29 open-ended questions, which required 4 to 6 min in total to complete. There were three segments in the overview poll. Section 1 collected the information associated with the respondent’s profile (13 items) such as name, age, gender, religion, domicile, educational qualification, profession, working experience, monthly salary, remittances, number of dependent people, and other sources of family income. Section 2 of the questionnaire (8 items) includes questions on the COVID-19 pandemic disruption of daily life, job status, salary reduction, and perception of the COVID-19 management by the government. The third section includes the questions (8 items) on psychological impact (depression, anxiety, and stress) during the lockdown due to the pandemic. The sample data of 180 Indian migrants in the Kingdom of Saudi Arabia belonging to different regions (states) were collected during the months of April and May 2021. The sample data consist of 98.8% male migrants and 1.2% female migrants. The majority of the working Indian migrants in the Saudi Arabia are male. According to UNDESA-2019, in total female migrants were around 30% of the total migrants; however, the majority of them are dependent and not working [32]. This paper includes the sample of only those migrants who were working, and therefore the sample of female migrants is smaller. A five-point Likert scale was used to collect the perception towards COVID-19 management by the government and a two-point Likert scale was used to assess psychological impact on migrants during the COVID-19 pandemic. The collected data were scrutinized through the ‘statistical package for social sciences’ (SPSS) version 28 (IBM, Armonk, NY, USA) and descriptive statistics were used for all covariates and survey responses.

## 5. Results, Findings, and Discussion

### 5.1. Demographic Profile of the Migrants

Table 1 illustrates the demographic profile of the surveyed migrants. It is depicted that the majority of Indian migrants in the Saudi Arabia (68.3%) were aged between 30 to 50. 15% were in between 20–30 and 16.7% were above 50 years of age. Migration to Saudi Arabia for work is mostly undertaken by males: around 98.8% were male migrant-workers and only 1.2% were female workers. The maximum number of the migrants (90.5%) belong to the religion of Islam (Muslims), 7.8% were Hindus, and 1.7% were Christians. Around 56.1% of the migrants belong to Uttar Pradesh (a state in India), 26.7% were from Bihar, 4.4% were from the National Capital of Delhi and Madhya Pradesh each, 2.8% were from Kerala, and only 5.6% were from the other states of India. The majority of the respondents (35.6%) hold bachelor’s degree (graduation), 26.7% hold an intermediate (senior secondary schooling) degree, 16.1% had studied until high school (10th class), 12.2% were post graduates, and only 2.2% hold a doctorate degree; however, 7.2% of the migrant workers were not educated.

### 5.2. Occupational Structure and Earnings of the Migrants

Table 2 presents the structure of employment, earnings, and remittances of Indian migrants in Saudi Arabia. Migration from India to Saudi Arabia consists of semi-skilled labor or unskilled labor. The majority of the surveyed respondents (24.4%) were engaged as technicians, 23.3% were sales workers, 10.6% were clerical support workers, 16.1% were plant and machine operators, and 12.2% were casual laborers (elementary activities). However, 10.6% were managers and professionals (skilled labor). Nearly half of the surveyed migrants (48.9%) earned less than SAR 2500 (Saudi Riyal) in a month and 42.8% were earning between SAR 2500 to 5000. Only 8.3% of the migrants were earning above SAR 5000; however, 91.7% were earning less than SAR 5000. The majority of the migrants (78.3%) did not have any other sources of income, however 21.7% had other sources of income from agriculture, rent, and income from businesses. India holds the highest number of international migrants and is the highest remittance receiving country in the world. The majority of the migrants (47.2%) remit less than SAR 1000, 32.8% were remitting between SAR 1000 to 3000, and 16.7% were sending between SAR 3000 to 5000; however, only 3.3% were remitting above SAR 5000 per month. The number of family members dependent on the remittances by the migrants varied: 51.6% of the migrants had 5 or more than 5 family members, 36.4% had 4 family members, and 12.3% had less than 3 family members.

### 5.3. Employment Status and Working Conditions during Lockdown (COVID-19)

**Figure 1:**
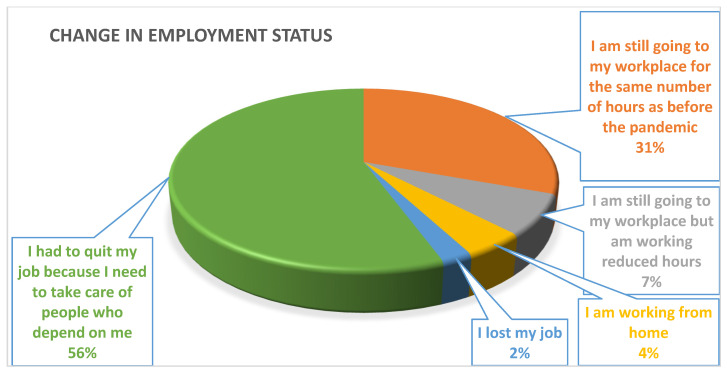
Changes in Employment Status during Corona Pandemic.

Table 3 and Figure 1 describe the employment status and working hours of the surveyed migrants. The majority of the migrants (50.6%) did not work during the lockdown period of the pandemic in Saudi Arabia; however, 32.8% of the respondents worked between 5 to 8 hours in day, 2.8% worked only 1 to 3 hours a day, 8.3% were working for 3 to 5 hours, and 5.5% of them worked more than 8 hours in a day. Lockdown had reduced the working hours of the migrants; hence the majority of the migrant workers were doing activities other than work. In total, 22.2% of the respondents spent majority of their time watching TV, 13.3% using social networking sites, 6.7% in telephonic conversations, 6.7% playing games, and 18.3% on other activities. Only 32.8% of the respondents were spending their maximum time on work.

The collected data for working experience reveal the fact that the number of new migrants is low: only 2.2% of the respondents had been working in Saudi Arabia for only one year, which overlaps with the era of the pandemic. In total, 34.5% of the respondents had been working for the last 2 to 4 years, 33.3% for 4 to 6 years, 13.9% for 6 to 8 years, and only 16.1% had been working over 8 years in the Kingdom of Saudi Arabia. Lockdown and travel restrictions in India and in Saudi Arabia restrict the movement of migrants. Only 8.3% of the migrants return back to India during the pandemic and 91.7% of migrants decided to stay in the destination country. The main reason for return back to India during the pandemic is the job losses and salary cuts. This pandemic not only changed working hours but also impacted the earnings of the migrant workers. Around 26.1% of the migrants had experienced losses in their average earnings between SAR 500 to 1000, 9.5% had lost between SAR 1000 to 2000, and 3.3% had lost above SAR 2000; however, 50.6% of the migrants lost below SAR 500. Loss in earning will directly affect the remittances. The majority of the migrants (58.3%) remit below SAR 1000 during lockdown period, 26.1% were remitting between SAR 1000 to 2000, 12.2% were sending between SAR 2000 to 3000, and only 3.4% were sending above SAR 3000 to India during the lockdown period. Employment status has drastically changed during the pandemic. The majority of the respondents (55.6%) quit their job because they needed to take care of their family, 30.6% of the respondents were still going to work for the same number of hours as before the pandemic, 7.2% were working reduced hours, 4.4% were working from home, and 2.2% of the respondents lost their job in the pandemic.

### 5.4. Changes in Remittances

**Figure 2:**
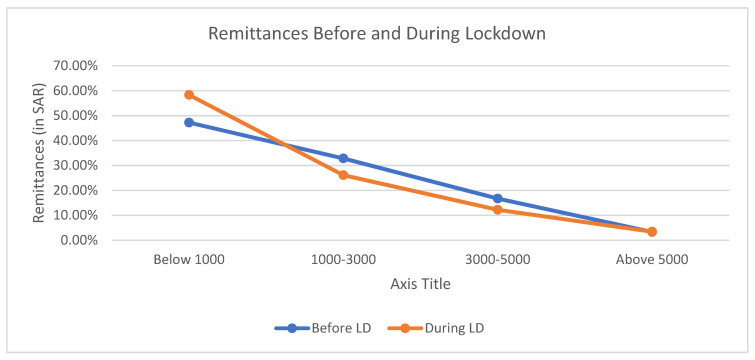
Change in remittances during the pandemic.

Figure 2 represents the changes in the distribution of remittances affected by the pandemic. The majority of the migrants were remitting below SAR 1000 before and after pandemic; however, before the pandemic, 47.2% of the migrants were remitting this amount but during the lockdown period of the pandemic that figure rose to 58.3%. This means that more migrants were remitting a lesser amount. The reduction in remittance was captured from those who were remitting either in between SAR 1000–2000 and 2000–3000 by 32.8% and 26.1%, respectively, before the pandemic, and 26.1% and 12.2% after pandemic. It was also found that remittance by higher income group tends to rise during the lockdown period of the pandemic. Before the pandemic, 3.3% of the migrants were remitting above SAR 5000 but during lockdown period the remittances in this category rose to 3.4%.

### 5.5. Dependent Sample t-Test

Dependent or paired sample *t*-test is used to compare the differences in the value of same sample at two different times. To test whether the comparison of remittances shown in Figure 2 is statistically significant, a *t*-test is applied. The null hypothesis was ‘there is no statistical difference in remittances before and during the lockdown of the pandemic’. The mean and standard deviation of remittances before lockdown were estimated to be 1.78 and 0.917, respectively, and during lockdown the mean = 1.62 and Std. Deviation = 0.885. Table 4 describe the results of the paired *t*-test. T statistics of 3.924 with 179 degree of freedom corresponded to the *p*-value = 0.000, which is less than 0.05; therefore, we reject the null hypothesis. It means that there is statistically a difference in the remittances before and during lockdown

### 5.6. Migrants’ Perception of the COVID-19 Management by the Government of Saudi Arabia

The researchers used six questions to assess respondents’ perceptions of the COVID-19 management by the government of Saudi Arabia. The responses were registered in a five-point Likert scale varying from ‘very good’ to ‘very poor’, as presented in Table 5. The migrants were found to be satisfied with the COVID protection related information provided by the government. In total, 51.1% responded ‘very good’, 46.1% responded as ‘good’, only 2.8% believed that ‘average’ information was provided, and none of the migrants responded ‘poor’ or ‘very poor’. Half of the migrants reported ‘very good’ and 48.9% responded ‘good’ in regard to safety measures taken by the Saudi government. Almost the same perception was found in the case of medical facilities provided by the government: 54.4% reported as very good, 44.4% as good, and only 1.1% were average. In response to whether migrants had faced any difficulties sending money to their family during the lockdown period, 51.1% reported ‘very good’, 42.8% reported ‘good’, 3.9% reported ‘average’, and only 2.2% reported ‘poor’ facilities to send money during the lockdown period. The researchers also asked about the food and other basic facilities provided by the government: 46.7% responded ‘very good’ and ‘good’ separately; however, 6.7% responded ‘average’. Furthermore, 46.1% of the respondents reported ‘very good’ living conditions, 49.4% responded ‘good’, and only 4.4% reported ‘average’ living condition during the lockdown period.

### 5.7. Migrants’ Perceptions of COVID-19 Management by Government of India

The researchers also tried to analyze the perceptions of the migrants towards the facility provided in the evacuation of migrants during the lockdown period. Two questions were asked of the respondents, and responses are presented in Table 6. The first question was related to the help provided by the embassy of India in Saudi Arabia: 33.9% of the respondents reported ‘very good’, 56.1% responded ‘good’, 7.2% responded ‘poor’, and 0.6% responded ‘very poor’. This question was relevant because some migrants may face the problem of visa expiry, passport renewal, or issues related to working contracts. The second question was related to the evacuation of the migrants through transport facilities: 56.1% of the respondent reported ‘very good’, 31.7% reported ‘good’, 8.3% reported ‘average’ and 3.9% responded ‘very poor’. This question was also relevant because the uncertainty which arises due to the spread of Corona virus forces migrants to return to India.

### 5.8. Combined Mean of Perceptions

Table 7 presents the combined mean of the perceptions towards COVID-19 management by the Government of India and Government of Saudi Arabia. A lower mean value indicates better perceptions. The combined mean of the perceptions towards government of Saudi Arabia is 1.54, which is less than the combined mean value 1.7 of the perception towards the government of India. This result shows that the government of Saudi Arabia managed COVID-19 better than the government of India according to migrants’ perceptions. However, this perception is based on few variables and the responses of the migrants available in Saudi Arabia during lockdown period.

### 5.9. Chi-Square Test

A chi-square test was applied to discover the association between perceptions of COVID-19 management by the government across the different professions/occupations. The sample data of occupational structure were classified as Professionals, Technicians, Clerical Support Workers, Service and Sales Workers, Elementary Occupation, Plant and Machine Operator, and others. The null hypothesis was ‘There is no significant difference in the opinion/perception of the migrant workers towards the COVID-19 management by the government across different occupations’, and the alternative hypothesis was ‘there is significant difference in the opinion/perception of the migrant workers towards the COVID-19 management by the government across different occupations. The relationship between these variables was found to be significant as calculated by chi-square value and *p*-value which is less than 0.05 (5% level of significance) as presented in the Table 8. Therefore, we reject the null hypothesis. It means that there was a significant difference in opinion of the migrant workers towards the COVID-19 management by the government of Saudi Arabia and India. Professionals, technicians, and elementary occupational workers were found to have low negative opinion towards COVID-19 management by the government, especially towards transport facilities provided by the government of India, help provided by the embassy of India, and facilities to send money from Saudi Arabia to India. However, clerical support workers, service and sales workers, and plant and machine operators had highly positive opinions concerning the COVID management by the government.

### 5.10. Comparing the Perceptions of Migrants with Other Citizens

Perceptions of the citizens or migrants may differ or be the same in different countries, depending upon the infrastructure and decisions taken by the government. Several studies were carried out to investigate the perceptions of the citizens or migrants in a country. For instance, a study on the perception of health care workers during the COVID-19 pandemic in the case of Saudi Arabia was conducted, which confirmed that the majority of the respondents (93.6%) were happy and felt safe in regard to the government decision of lockdown and 94.7% supported the travel restriction imposed by the government [33]. Similarly, our study also confirms that the majority of the migrants strongly agreed or agreed with the government decision. For instance, 97.2% of the migrants agreed with the information provided by Saudi government related to the protection from Corona virus. In total, 98.8% of the respondents were happy with the safety measures taken by the Saudi government. Another study was conducted to explore the perception of the public of the government of Singapore in relation to COVID-19 related information. The results of the study confirm that majority of the respondents (99.1%) agreed or strongly agreed on the COVID-19 related information provided by the government and 97.9% believed the Singapore news agency [34]. Another study was carried out in Bangladesh to explore the public perception of government measures related to COVID-19. The result of the sample survey reveals the fact that the majority of the respondents (58%) were not satisfied by the measures taken by the government of Bangladesh. However, 40% of the respondents were found to be satisfied with government decisions [35]. Our study reveals the fact that the majority of the migrants were satisfied by the decision taken by the Saudi government. Another study for Bangladesh was conducted and revealed the fact that the majority of the respondents (62%) strongly agree that the healthcare system was not able to handle the pandemic and 68.6% believe that the government of Bangladesh needs support from the public to handle the pandemic [36]. In our study, 98.8% of the respondents were happy with the medical facilities provided by the government of Saudi Arabia, 93.8% were satisfied with the facility to send money, 90% were happy with the help provided by the embassy of India, and 87.7% were satisfy with the transport facility provided by government of India during the lockdown period.

### 5.11. Mental Health Status of the Migrants

COVID-19 had not only influenced the economic and physical health of the people but also their mental status. This pandemic had drastically changed the mental status of the Indian migrant workers in Saudi Arabia. Table 9 describes the levels of anxiety, depression, and stress among the migrant workers. The majority of the migrants feel nervous (67.8%), depressed (63.3%), and lonely (72.2%) during the pandemic. It was also reported that 70% had difficulties in concentrating and 66.7% had a hard time in sleeping; however, the majority of them (91.7%) were feeling hopeful about the future, which shows silver lining at the end of the tunnel. It was also observed that only 2.2% of the migrants were Corona positive, 1.7% of their member households were Corona positive, and most of them (78.3%) were not scared of virus.

### 5.12. Comparison of Mental Health of the Migrants by Age, Domicile and Education

#### 5.12.1. Felt Nervous, Anxious, or on Edge

**Figure 3:**
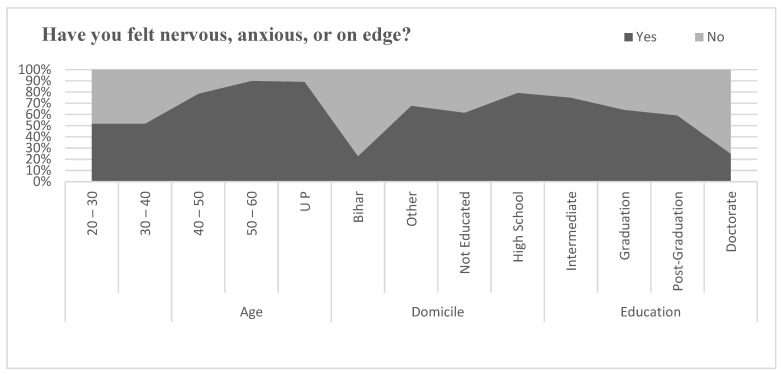
Comparison of feeling nervous.

Figure 3 describes feeling nervous, anxious, or on edge during pandemic by Indian migrants in Saudi Arabia. Around half of the young population aged between 20 to 40 felt nervous, and 78.5% of the migrants between the age of 40 to 50 and 90% of the age group above 50 felt nervous during the pandemic. The sample data reveal the fact that young migrants were less nervous than older migrants. The migrants from Bihar were found to be less nervous than Uttar Pradesh and other states of India. The nervousness of the migrants was also influenced by the levels of education of the migrants. Higher level of education implies a lower level of nervousness: 25% of doctorate, 59% of post-graduates, 64% of graduates reported nervousness during pandemic; however, 75% of intermediate, 79% of high school educated, and 61.5% of uneducated migrants reported nervousness. To investigate the relationship between nervous feeling by age, domicile, and education, correlation coefficient was applied. The result of correlation coefficient is shown in Table 10. Pearson’s correlation between felt nervous and age of the migrants was found to be negative and statistically significant (r = −0.311, *p* < 0.01). Similarly, the relationship between felt nervous and domicile of the migrants was found to be positive and statistically significant (r = 0.262, *p* < 0.01). However, the relationship between felt nervous and education level of migrants was found to be positive but statistically insignificant (r = 0.133, *p* > 0.01).

#### 5.12.2. Felt Depressed

**Figure 4:**
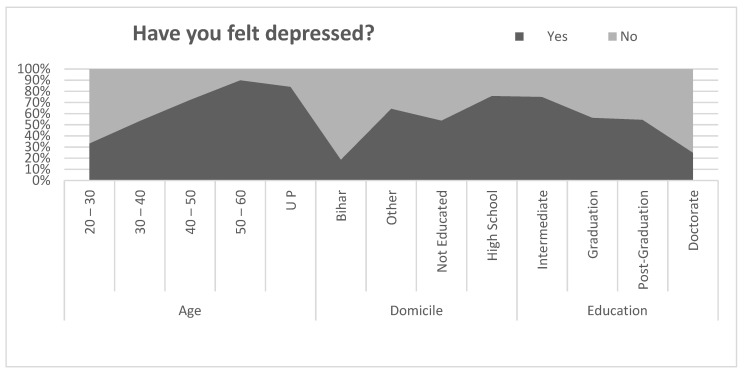
Comparison of feeling depressed.

Figure 4 represents the level of depression among the sample migrant workers during the pandemic. Less than half of the young migrants aged between 20 to 40 reported feeling depressed, however 72% of the migrants between the ages of 40 to 50 and 90% of the migrants aged above 50 reported feeling depressed during the pandemic. The sample data reveal that young aged migrants were less depressed than old age migrants. The 84.1% migrants from Uttar Pradesh reported feeling depression, however only 18.8% of the migrants from Bihar reported depression and 64.5% from other states were in depression during the pandemic. Lower levels of depression were reported by highly educated migrants (25% of doctorate, 54% of postgraduate, and 56% of graduate) and uneducated migrants (53%); however, 75% of the intermediate and high school educated migrants reported depression. To investigate the relationship between feelings of depression by age, domicile, and education, a correlation coefficient was applied. The result of the correlation coefficient is shown in Table 11. Pearson’s correlation between felt depressed and age of the migrants was found to be negative and statistically significant (r = −0.368, *p* < 0.01). Similarly, the relationship between felt depressed and the domicile of the migrants was found to be positive and statistically significant (r = 0.236, *p* < 0.01). However, the relationship between felt depressed and education level of migrants was found to be positive but statistically insignificant (r = 0.140, *p* > 0.01).

#### 5.12.3. Felt Lonely

**Figure 5:**
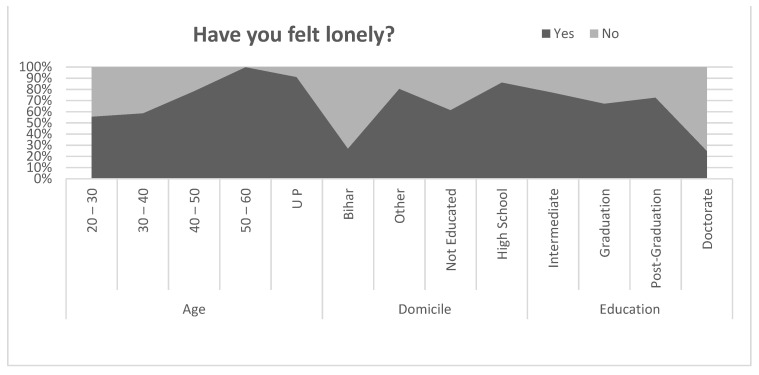
Comparison of feeling lonely.

Figure 5 shows the loneliness among the different types of the migrants. The migrants in the age group of below 40 reported less loneliness than the migrants above the age of 40. All migrants above the age of 50 reported that they felt lonely during the pandemic; however, 78.5% in the age group of 40 to 50, and around 56% of the age below 40 reported feeling loneliness during the pandemic. The migrants from Bihar were feeling less loneliness than the migrants from other states of India. The loneliness among differently educated migrants were not educated migrants (61.5%), high school (86.2%), intermediate (77.1%), graduation (67.2%), post-graduation (72.7%), and doctorate only 25%. To investigate the relationship between felt lonely by age, domicile and education, a correlation coefficient was applied. The result of the correlation coefficient is shown in Table 10. Pearson’s correlation between felt lonely and age of the migrants was found to be negative and statistically significant (r = −0.333, *p* < 0.01). Similarly, the relationship between felt lonely and domicile of the migrants was found to be positive and statistically significant at the level of 0.05 (r = 0.178, *p* < 0.05). However, the relationship between felt lonely and education level of migrants was found to be positive but statistically insignificant (r = 0.106, *p* > 0.01).

#### 5.12.4. Felt Hopeful about the Future

**Figure 6:**
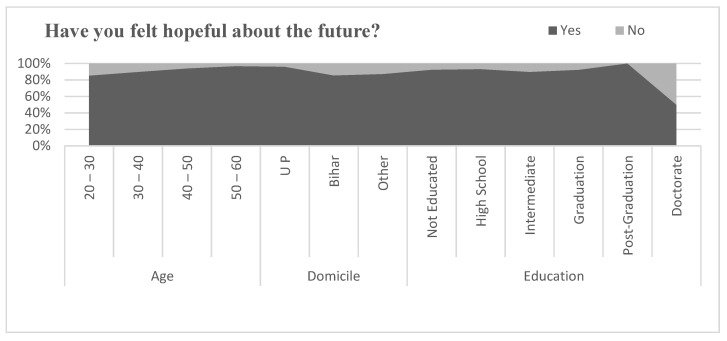
Comparison of feeling hopeful about future.

Figure 6 expresses the hopefulness about the future by migrant workers during the pandemic. The majority of all age groups of migrants, from all states and levels of education, reported hoping for better events to happen in the future; however, hopefulness in highly qualified migrants was reported to be less than in all other migrants. To investigate the relationship between felt hopeful about the future and age, domicile, and education, a correlation coefficient was applied. The result of correlation coefficient is shown in Table 10. Pearson’s correlation between felt hopeful about the future and age of the migrants was found to be negative but statistically insignificant (r = −0.132, *p* > 0.05). Similarly, the relationship between felt hopeful about the future and domicile of the migrants was found to be positive and statistically significant at the level of 0.05 (r = 0.165, *p* < 0.05). However, the relationship between felt hopeful about the future and education level of migrants was found to be positive but statistically insignificant (r = 0.027, *p* > 0.05).

#### 5.12.5. Difficulties in Sleeping and Concentration

**Figure 7:**
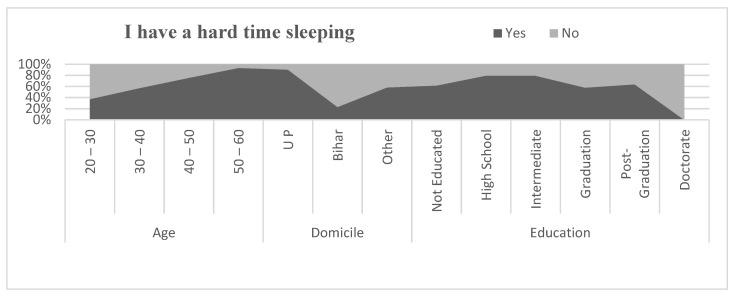
Comparison of hard time sleeping.

**Figure 8:**
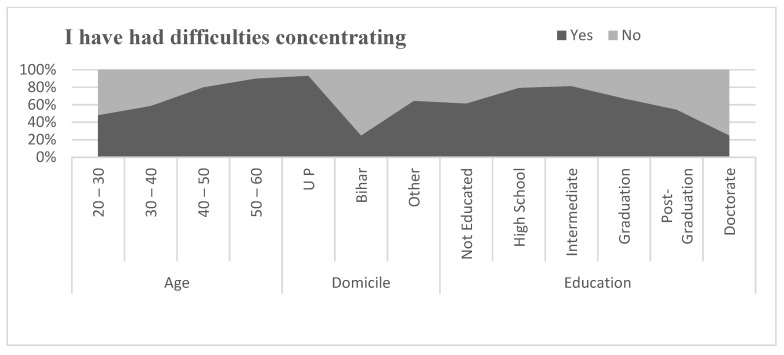
Comparison of having difficulties in concentration.

Figure 7 and Figure 8 describe the anxiety among migrant workers through sleeping problems and difficulty in concentrating. The problem of anxiety was reported as more severe in the age group of above 40 than the age group below 40. It was also seen that migrants who belongs to Bihar were found to have less of an anxiety problem than the migrants from other states. The problems of anxiety were also correlated to the level of education of the migrants. More educated migrants reported less anxiety than the lower educated migrants during the pandemic. To investigate the relationship between difficulty sleeping and difficulties in concentration by age, domicile, and education, a correlation coefficient was applied. The result of the correlation coefficient is shown in Table 10. Pearson’s correlation between difficulty sleeping and age of the migrants was found to be negative and statistically significant (r = −0.372, *p* < 0.01) and difficulties in concentration and age was also found to be negative and statistically significant (r = −0.315, *p* < 0.01). Similarly, the relationship between difficulty sleeping and domicile of the migrants was found to be positive and statistically significant (r = 0.320, *p* < 0.01) and the relationship between difficulties in concentration and domicile was also found to be positive and statistically significant (r = 0.289, *p* < 0.01). The relationship between difficulty sleeping and education level of migrants was found to be positive but statistically significant at the level of 0.05. (r = 0.175, *p* < 0.05) and the relationship between difficulties in concentration and education level of the migrants was also found to be positive and statistically significant at the level of 0.05 (r = 0.151, *p* < 0.05).

#### 5.12.6. Comparison of Mental Health Effect by Number of Family Members

Table 11 presents the comparison of mental health impacted by number of family members of the migrants. Sample data disclose the fact that number of family members is directly related to the psychological stress on migrants. Only 50% of migrants with family members below 5 felt nervous, 35.9% depressed, 53.1% lonely, 42.1% had a hard time sleeping, and 50% had difficulties in concentrating; however, 77.5% of migrants with family members equal to 5 or above felt nervous, 78.4% depressed, 82.8% lonely, 80.2% have a hard time sleeping, and 81.1% had difficulties in concentration. The majority of the migrants (87.5% with family member below 5 and 94% with family member equal to 5 or above) felt hopeful about the future. The psychological problems are severe in the case of the migrants above the age of 40 and migrants with higher number of family members, because of social responsibility and low capabilities to face interpersonal challenges. To test the hypothesis and investigate the relationship between these statements and the number of family members, a correlation coefficient was applied. The result of correlation coefficient is shown in Table 9. Pearson’s correlation between these statements and the number of family members of the migrants was found to be negative and statistically significant. However, the relationship between felt hopeful about the future and number of family member is positive but statistically insignificant (r = −0.143, *p* > 0.05).

#### 5.12.7. Comparing the Psychological Impact on Migrants before and during COVID-19

The results of the study confirm that 67.8% of the respondent migrants reported feeling nervous and 63.3% were depressed due to the COVID-19 pandemic. The level of psychological impact is much higher and severe among the migrant workers than before. One study was carried out to find out the prevalence of depression among migrant workers in AL-Qassim, Saudi Arabia, by taking a cross-sectional survey of 400 workers in 2016. The results of the study confirmed that 20% of the migrants reported the symptom of depression. It was also reported that level of depression varied by age but not by duration of stay [37]. Another similar study was conducted in 2011 to find out the prevalence of depression among migrant workers of UAE. The survey results of the 239 samples revealed that 25.1% of the migrants reported symptoms of depression. They also concluded that prevalence of depression is correlated with physical illness. In the same study, 6.3% of the respondents reported suicidal ideation and 2.5% had already attempted suicide [38]. Another study to find out the prevalence of depression among migrant workers was carried out in case of Qatar in 2016, which reported that 57.9% of the migrants had symptoms of depression [39]. Hence, it is clear that the percentage of migrants in our study which reported symptoms of depression and feeling nervous is much higher than the earlier studies; therefore, the COVID-19 pandemic had a severe psychological impact on Indian migrants working in Saudi Arabia.

## 6. Limitations

The main limitations of the study are that it focuses on the Indian male migrants in Saudi Arabia with a sample data of 180, however the sample of female migrants are very less. It also uses few tests and strategies. The current study does not emphasis the effect on migrant’s family in India during pandemic. Apart from it, this study does not consider COVID-19 vaccination process, problems and its impact on migrants in Saudi Arabia. The future scope of study in this area needed to analyze the economic and psychological impacts of COVID-19 on female migrants, migrant’s wife, children or family. In addition to it, the similar type of study can be done considering the female migrants. 

The policy implications from the findings of the research draw attention of the policy makers towards protective measures need to be implemented to save migrants during ongoing pandemic. The government should take some necessary steps such as financial benefit scheme to overcome the problems in the reduction of migrant earnings and remittances. Employment status of the migrants had drastically changed, which also drawing attention of policy makers to create more employment opportunities to reduce unemployment and underemployment. The government should not focus only on vaccination and physical fitness of the migrants but also need to find out the cure of the psychological impact arising during the pandemic.

## 7. Conclusions

The ongoing pandemic COVID-19 has severe economic and psychological effects on the world economy. This pandemic has changed the working environment and living style of the people across the world. This study analyzed the economic impact and health status of the Indian migrants in Saudi Arabia. The study revealed that majority of the migrants do not worked during the lockdown period and 42% of the migrants worked less than 8 hours in a day. Working experience of the migrants reveals a tendency is reduction in new international migration. Only 2.2% of the migrants reported work experience below one year, 35.5% were reported between 2 to 4 year and more than 60% of the migrants reported the work experience above 4 years.

During lockdown period migrants who were not working or working for less number of hours spent his maximum time on watching TV or surfing social networking sites. Majority of the migrants (48.9%) were earning below SAR 2500 (Saudi Riyal) and remitting below SAR 1000 in a month and majority of them were either 5 members family or more than 5. Around half of the migrants do not worked during the lockdown period and 42% of the migrants worked less than 8 hours in a day. Around 61% of the migrants reported an average loss in salary of below SAR 500 and 26% reported a loss between SAR 500 to 1000, however only 12% reported a loss above SAR 1000 during lockdown period of the pandemic. It has also been observed that number of migrants who were remitting below SAR 1000 had increased and remittances by middle income group were found to be decreased during lockdown period of the pandemic. The major reason of loss in salary and reduction in remittances were change in the employment status (reduced working hours, job loss, etc.) of the migrants. The perceptions of the migrants towards COVID management by the Saudi Arabia were found to be more positive than COVID management by the government of India. Chi-square test result showed there is significant difference in opinion of the migrant workers towards the COVID-19 management by the government of Saudi Arabia and India within different professions/occupations. The majority of respondents reported of feeling nervous, depressed, lonely, hard time sleeping and difficulties in concentrating, however majority of them also hopeful about the future. The problem of anxiety and depression were found to be more in the age group of above 40 than the age below 40. It was also been observed that migrants from Bihar were feeling less nervous, depressed and lonely than the migrants from other states of India. Not educated migrants were more affected of nervousness, depression and anxiety problem than the other migrants. The psychological problems are severe in case of the migrants above the age of 40 and migrants with higher number of family member, because of social responsibility and low capabilities to face interpersonal challenges.

## Figures and Tables

**Table 1:** Demographic Profile of the Migrants.

Variables	Levels	Data in Number	Data in %
Age	20–30	27	15.0%
	30–40	58	32.2%
	40–50	65	36.1%
	50–60	30	16.7%
Gender	Male	178	98.8%
	Female	2	1.2%
Domicile	Uttar Pradesh	101	56.1%
	Bihar	48	26.7%
	Delhi	8	4.4%
	Madhya Pradesh	8	4.4%
	Kerala	5	2.8%
	Other	10	5.6%
Education	Doctorate	4	2.2%
	Post-Graduation	22	12.2%
	Graduation	64	35.6%
	Intermediate	48	26.7%
	High School	29	16.1%
	Not Educated	13	7.2%

Source: Calculated by the authors from Google-Form questionnaire.

**Table 2:** Structure of Employment Status and Earnings.

Variables	Levels	Data in Number	Data in %
Profession/Occupation	Professionals	19	10.60%
	Technicians	44	24.40%
	Clerical Support Workers	19	10.60%
	Service & Sales Workers	42	23.30%
	Elementary Occupation	22	12.20%
	Plant & Machine Operator	29	16.10%
	Others	5	2.80%
Monthly Salary (in SAR)	Below 2500	88	48.90%
	2500–5000	77	42.80%
	5000–7500	6	3.30%
	7500–10,000	4	2.20%
	Above 10,000	5	2.80%
Remittances (in SAR)	Below 1000	85	47.20%
	1000–3000	59	32.80%
	3000–5000	30	16.70%
	5000–10,000	2	1.10%
	Above 10,000	4	2.20%
Number of Dependents	2	3	1.70%
	3	19	10.60%
	4	65	36.10%
	5	51	28.30%
	More than 5	42	23.30%
Does your family have other sources of income?	Yes	39	21.70%
	No	141	78.30%

Source: Calculated by the authors from Google-Form questionnaire. SAR: Saudi Riyal.

**Table 3:** Employment Status and Working Condition during Lockdown (COVID-19).

Statemenst	Levels	Frequency	Percentage
Number of daily working hour during lock down	Not Worked	91	50.60%
	1–3	5	2.80%
	3–5	15	8.30%
	5–8	59	32.80%
	Above 8	10	5.50%
Maximum time spent during lockdown	Work	59	32.80%
	Watching TV	40	22.20%
	Using social networking sites	24	13.30%
	Telephonic conversation	12	6.70%
	Playing games	12	6.70%
	Others	33	18.30%
For how many years you have been working in Saudi Arabia	Below 1 Year	4	2.20%
	2–4 Year	62	34.50%
	4–6 Year	60	33.30%
	6–8 Year	25	13.90%
	More than 8 Year	29	16.10%
Have you returned to India during lockdown?	Yes	15	8.30%
	No	165	91.70%
Average Salary loss during Corona (in SAR)	Below 500	110	61.10%
	500–1000	47	26.10%
	1000–2000	17	9.50%
	Above 2000	6	3.30%
Remittances during lockdown (in SAR)	Below 1000	105	58.30%
	1000–3000	47	26.10%
	3000–5000	22	12.20%
	Above 5000	6	3.40%

Source: Calculated by the authors from Google-Form questionnaire.

**Table 4:** Paired Sample *t*-Test.

Paired Sample *t*-Test								
95% Confidence Interval of the Difference								
	Mean	Std. Deviation	Std. Error Mean	Lower	Upper	t	df	*p*-Value
Remittances before lockdown—Remittances during lockdown	0.161	0.551	0.041	0.080	0.242	3.924	179	0.000

Source: Calculated by the authors from Google Form questionnaire.

**Table 5:** Perceptions of the migrants towards COVID-19 management by Government of Saudi Arabia.

Statement	Very Good	Good	Average	Poor	Very Poor	Total
COVID protection related information by Saudi Govt.	51.1%	46.1%	2.8%	NIL	NIL	100.0%
Safety measures taken by Saudi Government	50.0%	48.9%	1.1%	NIL	NIL	100.0%
Medical facility provided by Saudi Government	54.4%	44.4%	1.1%	NIL	NIL	100.0%
Facility to send money to India during lockdown	51.1%	42.8%	3.9%	2.2%	NIL	100.0%
Food and other facilities provided by Saudi Government	46.7%	46.7%	6.7%	NIL	NIL	100.0%
Living conditions in Saudi Arabia during lockdown	46.1%	49.4%	4.4%	NIL	NIL	100.0%

Source: Calculated by the authors from Google Form questionnaire.

**Table 6:** Migrant’s perceptions towards COVID management by Government of India.

Statements	Very Good	Good	Average	Poor	Very Poor	Total
Help provided by Embassy of India in Saudi Arabia	33.9%	56.1%	7.2%	2.2%	0.6%	100.0%
Transport facility provided by Government of India	56.1%	31.7%	8.3%	3.9%	NIL	100.0%

Source: Calculated by the authors from Google Form questionnaire.

**Table 7:** Combined mean of the perceptions.

Perceptions	N	Minimum	Maximum	Mean	Std. Deviation
Perception towards government of India	180	1	5	1.7	0.76
Perception towards government of Saudi Arabia	180	1	4	1.54	0.58

Source: Calculated by the authors from Google Form questionnaire.

**Table 8:** Chi-Square Analysis of Perceptions of COVID-19 Management by Govt. Within Different Professions/Occupations.

Perceptions	Chi Square Value	*p*-Value
COVID protection related information by Saudi Government	51.36	0.000
Safety measures taken by Saudi Government	29.18	0.004
Medical facility provided by Saudi government	21.64	0.042
Facility to send money to India during lockdown	39.44	0.002
Help provided by Embassy of India in Saudi Arabia	56.37	0.000
Transport facility provided by Governmentof India	40.14	0.002
Food & other facility provided by Saudi Government	28.86	0.004
Living condition in Saudi Arabia during lockdown	36.02	0.000

Source: Calculated by the authors from Google Forms questionnaire.

**Table 9:** Mental health of the migrants during pandemic.

Statements	Variables	Frequency	(%)
Have you felt nervous, anxious, or on edge?	Yes	122	67.80%
	No	58	32.20%
Have you felt depressed?	Yes	114	63.30%
	No	66	36.70%
Have you felt lonely?	Yes	130	72.20%
	No	50	27.80%
Have you felt hopeful about the future?	Yes	165	91.70%
	No	15	8.30%
I have a hard time sleeping because of the Corona	Yes	120	66.70%
	No	60	33.30%
I have had difficulties concentrating because of Corona	Yes	126	70.00%
	No	54	30.00%
Have you been tested Corona positive in Saudi Arabia?	Yes	4	2.20%
	No	176	97.80%
Are you scared of Corona virus?	Yes	39	21.70%
	No	141	78.30%
Does any of your family member infected of Corona virus?	Yes	3	1.70%
	No	177	98.30%

Source: Calculated by the authors from Google Forms questionnaire.

**Table 10:** Pearson’s correlation matrix.

**Statements**	**Age**	***p*-Value**
	**Pearson Correlation**	
Felt Nervous, Anxious, or on Edge	−0.311	0.000
Felt Depressed	−0.368	0.000
Felt Lonely	−0.333	0.000
Hard time sleeping	−0.372	0.000
Difficulties in concentration	−0.315	0.000
Hopeful about the future	−0.132	0.077
**Statements**	**Domicile**	***p*-Value**
	**Pearson correlation**	
Felt Nervous, Anxious, or on Edge	0.262	0.000
Felt Depressed	0.236	0.001
Felt Lonely	0.178	0.017
Hard time sleeping	0.320	0.000
Difficulties in concentration	0.289	0.000
Hopeful about the future	0.165	0.027
**Statements**	**Education**	***p*-Value**
	**Pearson correlation**	
Felt Nervous, Anxious, or on Edge	0.133	0.075
Felt Depressed	0.140	0.061
Felt Lonely	0.106	0.158
Hard time sleeping	0.175	0.019
Difficulties in concentration	0.151	0.044
Hopeful about the future	0.027	0.717
**Statements**	**Number of Family Member**	***p*-Value**
	**Pearson correlation**	
Felt Nervous, Anxious, or on Edge	−0.403	0.000
Felt Depressed	−0.464	0.000
Felt Lonely	−0.382	0.000
Hard time sleeping	−0.446	0.000
Difficulties in concentration	−0.429	0.000
Hopeful about the future	−0.143	0.055

**Table 11:** Comparison of psychological effect by number of family members.

Statements	Levels	Number of Family Member	
		Below 5	5 or More
Have you felt nervous, anxious, or on edge?	Yes	50.0%	77.5%
	No	50.0%	22.5%
Have you felt depressed?	Yes	35.9%	78.4%
	No	64.1%	21.6%
Have you felt lonely?	Yes	53.1%	82.8%
	No	46.9%	17.2%
Have you felt hopeful about the future?	Yes	87.5%	94.0%
	No	12.5%	6.0%
I have a hard time sleeping because of the orona	Yes	42.1%	80.2%
	No	57.9%	19.2%
I have had difficulties concentrating because of corona	Yes	50.0%	81.1%
	No	50.0%	18.9%

## Data Availability

The data used to support the findings of this study are available from the corresponding author upon request.
